# Agricultural Jiaosu: An Eco-Friendly and Cost-Effective Control Strategy for Suppressing *Fusarium* Root Rot Disease in *Astragalus membranaceus*

**DOI:** 10.3389/fmicb.2022.823704

**Published:** 2022-03-31

**Authors:** Youhui Gao, Yue Zhang, Xiaoqian Cheng, Zehui Zheng, Xuehong Wu, Xuehui Dong, Yuegao Hu, Xiaofen Wang

**Affiliations:** ^1^College of Agronomy and Biotechnology, China Agricultural University, Beijing, China; ^2^Biotechnology Research Institute, Beijing Academy of Agriculture and Forestry Sciences, Beijing, China; ^3^Biology Institute, Qilu University of Technology, Shandong Academy of Sciences, Jinan, China; ^4^College of Plant Protection, China Agricultural University, Beijing, China

**Keywords:** Agricultural Jiaosu, antifungal activity, *Astragalus membranaceus*, *Fusarium* root rot, sustainable agriculture

## Abstract

Root rot caused by the pathogenic fungi of the *Fusarium* genus poses a great threat to the yield and quality of medicinal plants. The application of Agricultural Jiaosu (AJ), which contains beneficial microbes and metabolites, represents a promising disease control strategy. However, the action-effect of AJ on *Fusarium* root rot disease remains unclear. In the present study, we evaluated the characteristics and antifungal activity of AJ fermented using waste leaves and stems of medicinal plants, and elucidated the mechanisms of AJ action by quantitative real-time PCR and redundancy analysis. The effects of AJ and antagonistic microbes isolated from it on disease suppression were further validated through a pot experiment. Our results indicate that the AJ was rich in beneficial microorganisms (*Bacillus*, *Pseudomonas*, and *Lactobacillus*), organic acids (acetic, formic, and butyric acids) and volatile organic compounds (alcohols and esters). It could effectively inhibit *Fusarium oxysporum* and the half-maximal inhibitory concentration (IC_50_) was 13.64%. The antifungal contribution rate of the microbial components of AJ reached 46.48%. Notably, the redundancy analysis revealed that the *Bacillus* and *Pseudomonas* genera occupied the main niche during the whole inhibition process. Moreover, the abundance of the *Bacillus*, *Pseudomonas*, and *Lactobacillus* genera were positively correlated with the pH-value, lactic, formic and butyric acids. The results showed that the combined effects of beneficial microbes and organic acid metabolites increased the efficacy of the AJ antifungal activity. The isolation and identification of AJ’s antagonistic microbes detected 47 isolates that exhibited antagonistic activities against *F. oxysporum in vitro*. In particular, *Bacillus subtilis* and *Bacillus velezensis* presented the strongest antifungal activity. In the pot experiment, the application of AJ and these two *Bacillus* species significantly reduced the disease incidence of *Fusarium* root rot and promoted the growth of *Astragalus*. The present study provides a cost-effective method to control of *Fusarium* root rot disease, and establishes a whole-plant recycling pattern to promote the sustainable development of medicinal plant cultivation.

## Introduction

Medicinal plants are important for human health and disease management. However, their consecutive monocultures have led to a very serious decline in soil quality and to an increase in soil-borne diseases (Wu et al., 2019). Root rot, caused by fungi of the *Fusarium* genus, is the most destructive soil-borne disease in monoculture systems (Klein et al., 2016; Tao et al., 2020), especially affecting the yield and quality of traditional Chinese herbs whose root and rhizomes are harvested for consumption, such as, *Astragalus membranaceus*. This plant, known as Huangqi in China, is one of the most widely used traditional Chinese herbs, and has been commonly infected by root rot caused by *Fusarium* spp. in recent years (Fu et al., 2014; Li et al., 2021). Crop rotation (Wang et al., 2015; Xiong et al., 2016), resistant cultivars (Pinaria et al., 2010), fungicides (Dignam et al., 2016) and soil fumigation (Gao et al., 2019) can control the pathogen to a certain extent. However, these measures are often impractical to apply to perennial medicinal plants because of their expensive, time consuming, and involve excessive labor-costs, and also because of their negative impact on food security and on the environment. At present, the control of soil-borne diseases should not focus only on eliminating pathogens, but also on regulating the microbial ecosystem. From the perspective of ecological balance, social security, and control efficiency, biological agents represent the optimal method to control *Fusarium* root rot (Liu et al., 2021). Many antagonistic microorganisms isolated from disease suppressive soils or rhizosphere soils of healthy plants (Dignam et al., 2016), such as *Bacillus* spp. (Khan et al., 2018; Wang et al., 2020), *Pseudomonas* spp. (Luo et al., 2019), *Lactobacillus* spp. (Magnusson et al., 2003), and *Penicillium* spp. (Usman et al., 2013), have been proved to be effective in disease suppression. However, these potentially antagonistic microbes have not been widely employed to control *Fusarium* root rot in medicinal plants, due to their instability and high-cost (Xiong et al., 2017). Therefore, a more stable, efficient, and low-cost control strategy is needed. A promising method is the application of Agricultural Jiaosu (AJ), a mix of beneficial microbes combined with metabolites.

The AJ is a microbial ecosystem produced through fermentation using organic waste as substrate (Zhang et al., 2020). It is widely used in agricultural production in China, especially in organic farming system, due to its low cost, stability, and simple operational. Previous studies have shown that AJ contains beneficial microorganisms, such as *Lactobacillus*, *Bacillus*, *Pseudomonas*, and *Pichia* spp., and organic acids, such as lactic and acetic acids (Arun and Sivashanmugam, 2015, 2017; Rahman et al., 2020). A number of studies reported that the application of AJ led to an increased biomass and quality of plants (Zhu et al., 2020), an increase in soil nutrients (Tong and Liu, 2020) and a change in the diversity of soil microbes (Wei et al., 2020). Moreover, our previous studies found that AJ can effectively inhibit soil-borne fungal pathogens for a long time (Zhang et al., 2020). However, the mechanisms underlying the AJ-induced disease suppression remain unclear. At the same time, it is also urgent to take measures to strengthen the antifungal activity of AJ, such as the optimization of fermented materials. Medicinal plants, especially those with anti-inflammatory properties, contain natural antifungal compounds. Their waste leaves and stems are considered as high-quality organic materials for AJ fermented because of their unique medicinal ingredients and easy decomposition.

In this study, *Astragalus membranaceus* was used as model crop and *Fusarium oxysporum* as model pathogen to reveal the mechanism of action of AJ and its effects on the suppression of *Fusarium* root rot. For this purpose, (1) the microbial and physicochemical properties of AJ fermented using the waste leaves and stems of medicinal plants were determined. (2) The antifungal activity of AJ against *F. oxysporum in vitro* was evaluated, and the relationships between beneficial microbes and physicochemical factors during the inhibition process were explored. (3) The main antagonistic microorganisms of AJ were isolated and their antifungal activity was evaluated *in vitro*. (4) The ability of AJ and antagonistic strains to suppress *Fusarium* root rot was verified *in vivo*. The aim of this study was to reveal the biocontrol mechanism of AJ, and may be useful to support this promising strategy for the control of soil-borne diseases in the future.

## Materials and Methods

An overview of the study methods is shown in Supplementary Figure 1. AJ preparation and characteristic detection methods were detailed presented in Sections “Agricultural Jiaosu Preparation and Pathogen Culture” and “Characterization of Agricultural Jiaosu.” Design and methods of AJ’s antifungal activity and biocontrol mechanism investigation were introduced in Sections “*In vitro* Antifungal Activity of Agricultural Jiaosu Against *F. oxysporum*” and “Dynamic Changes in Pathogen, Antagonistic Microorganisms, Organic Acids, and pH-Value During the Agricultural Jiaosu Inhibition Process.” Methods of screening AJ’s antagonistic strains in Section “Screening and Combination Effect of Antagonistic Strains Against *F. oxysporum*.” Design and methods for pot experiment in Section “*In vivo* Antifungal Assay of Agricultural Jiaosu and Antagonistic Strains by Greenhouse Pot Experiment.”

### Agricultural Jiaosu Preparation and Pathogen Culture

The main organic material used for AJ preparation consisted in the leaves and stems of medicinal plants, which included *Astragalus membranaceus*, *Radix scutellariae*, *Radix Sophorae flavescentis*, and *Bupleurum chinense*. The plant materials were collected in Lingqiu County, Datong City (located in Shanxi Province, China), and were cut into segments of 1–2 cm. Then, they were mixed together with brown sugar and deionized water at a ratio of 3:1:10 in a 5 L airtight glass container, and were kept at 35°C for 3 months to allow anaerobic fermentation. The mixed materials presented a pH of 6.29 and the following acid concentrations: lactic acid, 5.65 g/L; formic acid, 2.64 g/L; acetic acid, 4.55 g/L; propionic acid, 3.03 g/L; butyric acid, 0.50 g/L (the detection methods are shown in Section “Physicochemical Analysis”). After 3 months, the supernatant was used for subsequent experiments (Jiang et al., 2021).

The fungal pathogen *F. oxysporum* was isolated from the roots of an infected *A. membranaceus* plant (collected in Datong City, Shanxi Province, China) using a *Fusarium*-selective medium. The pathogen was cultured on PDA medium, added with 150 mg/L streptomycin, in the dark at 25°C for up to 5 days prior to use.

### Characterization of Agricultural Jiaosu

After 90 days of fermentation, the characteristics of AJ were evaluated based on two aspects, (I) the microbial community structure of AJ (including both bacterial and fungal), and (II) the physicochemical properties, including pH, organic acids and volatile organic compounds (VOCs). Three replicates (one sample for each replicate) were performed for each experiment.

#### Microbiological Analysis

Microbial DNA was extracted using the OMEGA Soil DNA Kit (M5635-02) (Omega Bio-Tek, Norcross, GA, United States). The quantity and quality of the extracted DNA was measured using a NanoDrop NC 2000 spectrophotometer (Thermo Fisher Scientific, Waltham, MA, United States) and agarose gel electrophoresis, respectively. The primers used for bacteria were 338F (5′-ACTCCTACGGGAGGCAGCA-3′) and 806R (5′-GGACTACHVGGGTWTCTAAT-3′), and those used for fungi were ITS1F (CTTGGTCATTTAGAGGAAGTAA) and ITS2 (GCTGCGTTCTTCATCGATGC). PCR program: 98°C 3 min, 98°C 30 s 25 cycles, 53°C 30 s, 72°C 45 s, and 72°C 5 min by a thermocycler PCR system. Illumina Miseq sequencing were conducted as described by Xu et al. (2021). Community structure was analyzed at the phylum and genus levels using the SILVA Release 132 database^1^ (Zhao et al., 2017) and UNITE Release 8.0 database^2^ (Koljalg et al., 2013).

#### Physicochemical Analysis

The pH values of AJ were determined by a micro-pH meter (Mettler-Toledo, Greifensee, Switzerland). The concentrations of organic acids (formic, acetic, lactic, and propionic acids) were analyzed using a high performance liquid chromatography (HPLC) system equipped with an ion-exchange column (Aminex HPX-87H; 300 mm × 7.8 mm, Bio-Rad Laboratories, Hercules, CA, United States) and a diode array detector (SPD-M20A, Shimadzu, Kyoto, Japan). The details of the chromatographic procedure and conditions are described in Cai et al. (2018).

The AJ’s VOCs were determined by headspace solid-phase microextraction coupled to gas chromatography mass spectrometry (HS-SPME/GC-MS) (Sanchez-Palomo et al., 2005). Briefly, the SPME fiber coatings used in this study is 50/30 μm PDMS/CAR/DVB (2 cm). 8 ml of AJ was placed it into a 15 mL extraction bottle, and 2.5 g NaCl was added to improve the extraction efficiency (Butkhup et al., 2011). Fibers were then exposed to the headspace of the extraction bottle for 40 min at 80°C. After extraction, the SPME fiber was inserted into the hot injector port (at 250°C) of the GC-MS system for 5 min where the extracted chemicals were desorbed thermally and transferred directly to the analytical column. The GC-MS analysis was performed on an Agilent gas chromatograph (model 7890A; Shiyanjia Lab, Zhejiang, China) coupled to a mass selective detector (model 5975C). Compounds were separated on a HP-5MS capillary column (30.0 m × 250 μm i.d.; 0.25 μm film thickness); the GC-MS conditions are described in Zhao et al. (2021). The compounds were identified based on the match of their GC retention times, retention indices and mass spectrum with the NIST11 library (Mohamad et al., 2018). The peak area of each compound was normalized using the area normalization method, and the relative abundance of each compound was calculated.

### *In vitro* Antifungal Activity of Agricultural Jiaosu Against *F. oxysporum*

#### Effect of Agricultural Jiaosu Dosage on the Antifungal Activity Against *F. oxysporum*

The antifungal activity of AJ was assessed through the agar plate diffusion method as previously described (Zhang et al., 2020). Briefly, PDA medium containing different AJ concentrations (5, 10, 15, 25, 35, and 50%, respectively) was prepared with AJ supernatant. The PDA medium without AJ was used as blank control (CK). Three replicates were performed for each treatment. After the medium solidified, a plug with a diameter of 6-mm containing *F. oxysporum* mycelium, which was obtained from a 5-day-old PDA culture, was placed at the center of each plate. These plates were incubated for 5 days in the dark at 25°C. The growth of the plug was measured every 24 h. The inhibition rate was calculated using the following formula (Aeron et al., 2011; Mohamad et al., 2018):


(1) $$\mathrm{Inhibition}\mathrm{rate}\left(\%\right)=\frac{\left(\mathrm{Fck}-\mathrm{Ft}\right)}{\left(\mathrm{Fck}-\mathrm{F0}\right)}\times 100$$

where F*ck* and F*t* represent the colony diameter of the fungal mycelium in the control and treatment, respectively, and F*0* is the diameter of the test fungus agar disks (6 mm).

In order to better measure the effectiveness of AJ, its half-maximal inhibitory concentration (IC_50_) was calculated. Briefly, taking the concentration of AJ as the *x*-value and the colony diameter of the pathogen at 120 h as the *y*-value, a quadratic regression analysis was carried out to obtain the fitting curve and polynomial. Then, half of the colony diameter of CK was considered as the *y*-value and was brought into the equation to calculate the corresponding *x*-value, which is the IC_50_ of AJ against *F. oxysporum* (Zhang et al., 2020).

#### Effect of the Microbial Components of Agricultural Jiaosu on the Antifungal Activity Against *F. oxysporum*

The inhibitory effect of the AJ’s microbial components against *F. oxysporum* was evaluated. Three different treatments were applied as follows: AJ, added 10% AJ; sterilized-AJ, added 10% sterilized AJ; CK, no AJ. Each treatment was repeated three times. According to the above-method in Section “Effect of Agricultural Jiaosu Dosage on the Antifungal Activity Against *F. oxysporum*,” the inhibition rate was determined after 5 days, and the contribution rate of different components to the inhibition of the pathogen was calculated using the following formula:


(2) $$\text{OCR}\%=\frac{\text{IR}\,s}{\text{IR}}\times 100$$

(3) MCR%=1-OCR

where OCR is contribution rate of other components, IR*s* is the inhibition rate of the sterilized-AJ treatment, IR is the inhibition rate of the AJ treatment, and MCR is the contribution rate of microbial components.

### Dynamic Changes in Pathogen, Antagonistic Microorganisms, Organic Acids, and pH-Value During the Agricultural Jiaosu Inhibition Process

To reveal the biocontrol mechanism of AJ during the inhibition process, an antifungal assay was carried out in potato dextrose broth (PDB) medium with the following treatments: AJ, 10% AJ treatment; and CK, without AJ as a control. The same amount of *F. oxysporum* mycelium block (6 mm diameter) was inoculated in each treatment (three mycelium blocks/100 mL). Three independent biological replicates were used for each treatment. The samples were collected at 0, 12, 24, 36, 48, 60, and 72 h to determine the pH-value and the concentrations of organic acids. Here, *Lactobacillus*, *Bacillus*, and *Pseudomonas* genera were selected as representatives of AJ’s antagonistic microorganisms, and their gene concentrations were determined by quantitative real-time PCR (qPCR).

The pH-value and organic acids were measured using the methods mentioned above in Section “Physicochemical Analysis.” The abundances of *F. oxysporum*, *Lactobacillus*, *Bacillus*, and *Pseudomonas* communities were quantified with the EF1H/EF2T (O’Donnell et al., 1998), F_alllact_IS/R_alllact_IS (Haarman and Knol, 2006), BacF/BacR (Mori et al., 2004), and Ps-for/Ps-rev primers (Garbeva et al., 2004), respectively (Supplementary Table 1); qPCR was carried out with the Applied Biosystems 7500 qPCR System (Applied Biosystems, CA, United States) by using the SYBR Green I fluorescent dye detection in 20-μL volumes containing 10 μL SYBR real-time PCR premixture (2×), and 0.4 μL of both forward and reverse primers (Wei et al., 2018). The PCR protocol was set as follows: 95°C for 5 min; followed by 40 cycles at 95°C for 15 s and 60°C for 30 s. The copy number of thee target genes was calculated based on previously described by Zhou and Wu (2018).

### Screening and Combination Effect of Antagonistic Strains Against *F. oxysporum*

#### Isolation and Identification of Antagonistic Strains

In order to identify the main antagonistic strains of AJ, the culturable bacteria and fungi were isolated using beef extract peptone agar medium and PDA medium containing 150 mg/L streptomycin. Bacteria and fungi were both incubated in the dark: the former at 35°C for 2–3 days, and the latter at 25°C for 3–4 days. These isolated bacteria and fungi were named from B1 to Bn, and from F1 to Fn, respectively. All microbial isolates were tested for their ability to inhibit fungal growth using a dual culture assay, following the method described in a previous study (Sun et al., 2021). The inhibition rate of the microbial isolates was calculated using the method described in Section “Effect of Agricultural Jiaosu Dosage on the Antifungal Activity Against *F. oxysporum.*”

The above assays detected seven isolates with high inhibition levels against *F. oxysporum*, their total genomic DNA was extracted using the Ezup Column Bacteria Genomic DNA Purification Kit (Sangon, Shanghai, China), following the manufacturer’s instructions. The 16s rRNA was amplified using the universal bacterial primers 27F (5′AGAGTTTGATCCTGGCTCAG-5′) and 1492R (5′-GGTTACCTTGTTACGACTT-3′). The amplicon was purified and sequenced by Sangon Biological Engineering Co., Ltd. (Shanghai, China) (Tamegai et al., 1997; Rameshkumar and Nair, 2009). The generated sequences were submitted to the NCBI GenBank, and were compared with gene sequences published in the NCBI website using the BLAST algorithm (Altschul et al., 1990). Distances were calculated according to standard parameters (Kumar et al., 2016), and phylogenetic trees were inferred using the neighbor-joining method with MEGA 7.0 (Saitou and Nei, 1987).

#### Antifungal Effect of Antagonistic Strain Combination Against *F. oxysporum*

Among the seven bacterial strains with the highest antagonistic ability, five were identified as *Bacillus* spp. and two as *Lysinibacillus* spp. Their antagonistic activity against *F. oxysporum* was examined by agar plate diffusion assay, including B1, B20, B23, B44, B50, B30, B38, 2L (equal proportion combination of the two *Lysinibacillus* isolates), 5B (equal proportion combination of the five *Bacillus* isolates), 2L + 5B (equal proportion combination of the seven bacterial isolates). Firstly, the seven strains were grown in beef extract peptone broth and were incubated at 35°C for 48 h, then, the 200 μL bacterial suspensions (isolated independently or in combination) were evenly spread on the surface of PDA plates, and a 6-mm plug of actively growing *F. oxysporum* mycelium was placed at the center of these plates containing the bacterial suspensions. Plates containing only sterile water were used as controls. Each treatment was repeated three time. All treatments were incubated in the dark at 25°C for 5 days. The mycelium growth of the pathogen was determined by measuring the colony diameter. The inhibition rate of the microbial isolates was calculated using the method described in Section “Effect of Agricultural Jiaosu Dosage on the Antifungal Activity Against *F. oxysporum.*”

### *In vivo* Antifungal Assay of Agricultural Jiaosu and Antagonistic Strains by Greenhouse Pot Experiment

The capacity of AJ and antagonistic strains to control *Astragalus* root rot was evaluated *in vivo*. The pot experiments with *Astragalus* were performed using the following five treatments: CK, not inoculated with *F. oxysporum* or biological agents; F, each plant inoculated with 1 mL of *F. oxysporum* suspension (10^5^ CFU/mL); F + AJ, each plant inoculated with 1 mL of *F. oxysporum* suspension (10^5^ CFU/mL) and 1 mL of 10% AJ; F + B1, each plant inoculated with 1 mL of *F. oxysporum* (10^5^ CFU/mL) and 1 mL of *B. velezensis* suspensions (10^6^ CFU/mL); F+B20, each plant inoculated with 1 mL of *F. oxysporum* (10^5^ CFU/mL) and 1 mL of *B. subtilis* suspensions (10^6^ CFU/mL). Each treatment included three replicates (pots) with twenty *Astragalus* seedlings per replicate. *Astragalus* seeds [*Astragalus membranaceus* (Fisch.) Bge. var. *mongholicus* (Bge.) Hsiao] were surface-sterilized with NaClO (3%; v: v) for 5 min and washed five times in sterile distilled water, and were then germinated on moist filter paper at 25°C in the dark (Wei et al., 2019). After 3 days, the germinated seeds were transferred to polyethylene plastic pots (100 mm × 150 mm × 120 mm) filled with 800 g of nutritional soil. The pots were randomly placed in a greenhouse at 28°C ± 2°C, 60–70% relative humidity, and a 16-h light/8-h dark cycle for 6 weeks until the end of the experiment (Chen et al., 2018).

After 2 weeks, all treatments—except for the CK treatment—were inoculated with *F. oxysporum* via root irrigation (Gao et al., 2019), and the control was treated with an equal volume of sterile PDA medium. The biocontrol agents were inoculated immediately after pathogens inoculation in the F + AJ, F + B1, and F + B20 treatments. Subsequently, biocontrol agents were applied once every 7 days for a total of three applications. The CK and F groups were treated with an equal volume of sterile water as controls. Plant characteristics were evaluated 4 weeks after pathogen inoculation, and they included: plant height, chlorophyll content (SPAD-502, Minolta), root length, root diameter, fresh weight and dry weight. The disease incidence was calculated based on the following formula (Zhai et al., 2021):


(4) $$\mathrm{Disease}\mathrm{incidence}\left(\%\right)=\frac{\mathrm{the}\,\mathrm{number}\,\mathrm{of}\,\mathrm{diseased}\,\mathrm{plants}}{\mathrm{total}\,\mathrm{number}\,\mathrm{of}\,\mathrm{investigated}\,\mathrm{plants}}\times 100$$

### Statistical Analyses

Statistical significance (ANOVA) was analyzed in SPSS 23.0 (IBM Corporation, NY, United States) and was established at a *P*-value less than 0.05. The figures were produced using Origin 2018 (OriginLab Corporation, Northampton, MA, United States), Excel 2019 (Microsoft Corporation, Seattle, WA, United States) and Adobe illustrator CC2019 (Adobe, San Jose, CA, United States). The analysis of the microbial community structure, and redundancy analysis were performed on the Personal Genes cloud Platform, freely available online.

## Results

### Characteristics of Agricultural Jiaosu

#### Microbial Community

**FIGURE 1:**
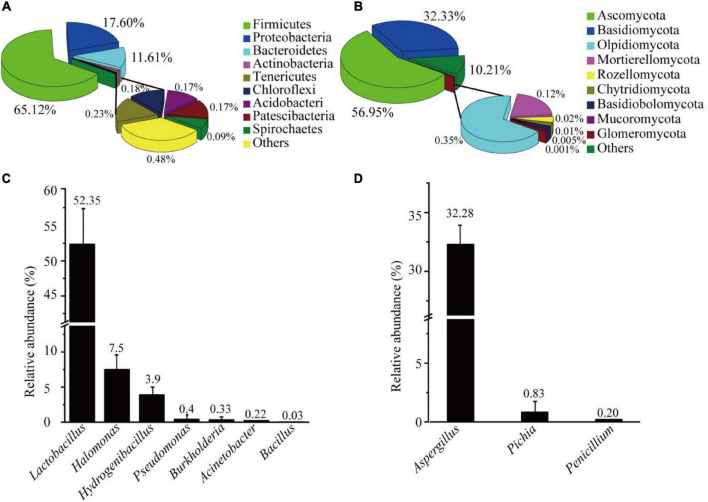
Microbial community structure of AJ: **(A)** bacterial community at the phylum level; **(B)** fungal community at the phylum level; **(C)** relative abundance of bacteria with potential biological control functions; **(D)** relative abundance of fungi with potential biological control functions. Values in the bar plot are expressed asmean ± standard deviation (*n* = 3).

The AJ’s bacterial and fungal communities were analyzed through the Illumina sequencing of 16S rRNA and ITS gene amplicons, which obtained a total of 4,262 bacterial ASVs and 587 fungal ASVs (Supplementary Table 2). Bacterial and fungal good’s coverage indexes were 0.9975, and 0.9998, respectively. Chao1, Simpson, Shannon and Pielou’s evenness indexes were higher for bacteria than for fungi (Supplementary Table 3). The results showed that the diversity, richness, and evenness of AJ’s bacteria were higher than those of fungi. Further analysis showed that the phyla representing more than 1% of the bacteria in the AJ included Firmicutes (65.12%), Proteobacteria (20.90%), Bacteroidetes (11.61%), and Actinobacteria (1.03%) (Figure 1A). Among the fungal community, the majority of species belonged to the phyla Ascomycota (56.95%) and Basidiomycota (32.33%) (Figure 1B). *Lactobacillus* and *Aspergillus* with potential biocontrol functions were the most abundant bacterial and fungal genus, accounting for 52.35, and 32.28% of the total, respectively (Supplementary Table 4). Moreover, other genera with potential biocontrol functions were also identified in the microbial community, such as *Pseudomonas*, *Bacillus*, *Burkholderia*, *Pichia*, and *Penicillium* (Figures 1C,D).

#### Physicochemical Properties

The AJ fermentation for 90 days, the pH value of AJ was 3.26 ± 0.01. The main organic acid was acetic acid, with a concentration of 13.08 ± 0.67 g/L. The concentration of formic, propionic, and butyric acids were 0.02 ± 0.00 g/L, 0.96 ± 0.19 g/L, and 0.63 ± 0.01 g/L, respectively. Lactic acid was not detected. Moreover, a total of 29 VOCs were detected by GCMS analysis (Supplementary Table 5), including 8 alcohols, 4 phenols, 11 esters, 2 hydrocarbons, 2 ketones, 1 ether and 1 amine. Esters and alcohols were the main compound types, accounting for 63.88% of the 29 detected compounds. It was concluded that 3-heptanol, 6- methyl-, phenol, 4-ethyl-2- methoxy-, L-.alpha.-terpineol, 1-Octen-3-ol, methyl salicylate, and phenol, 2-methoxy-6-(2-propenyl)- were the main VOCs in the AJ, accounting for 14.00, 9.07, 7.85, 7.61, 6.84, and 4.80%, respectively.

### Antifungal Activity of Agricultural Jiaosu Against *F. oxysporum*

**FIGURE 2:**
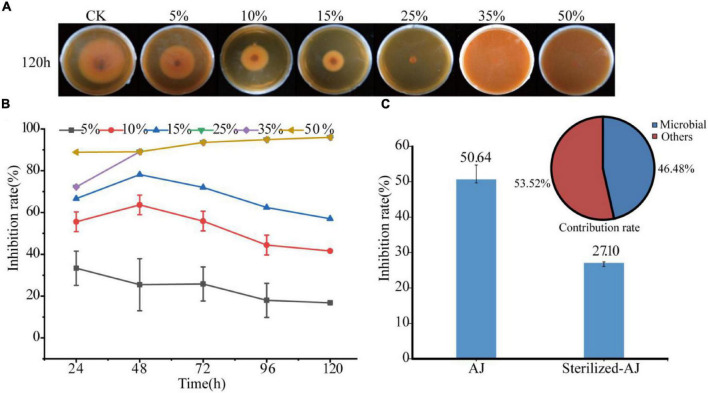
Antifungal activity: **(A)** antifungal test photos taken at 120 h; **(B)** inhibition rate of different dosages of AJ at different times; **(C)** inhibition rate and contribution rate of different AJ components. AJ, 10% AJ; sterilized-AJ, 10% sterilized AJ. Data are mean ± standard deviation, *n* = 3.

The inhibitory effect of the AJ dosage was tested against *F. oxysporum* as shown in Figure 2A. The higher the dose of AJ, the stronger the inhibition of *F. oxysporum*. When the dosage was higher than 25%, the growth of the pathogen was almost completely inhibited and the inhibition rate reached up to 95.97% at 120 h. The 5, 10, and 15% AJ treatments had the strongest inhibitory effect at 48 h, and the inhibition rates were 25.45, 63.64, and 78.18%, respectively (Figure 2B). The IC_50_ was calculated based on the above experimental data. The fitting formula obtained was: *y* = 0.0328x^2^ − 2.6289x + 57.59, *R*^2^ = 0.9851; and the IC_50_ of AJ against *F. oxysporum* was 13.64% (Supplementary Figure 2). Notably, when the inhibitory effect of different AJ components was tested, it was found that the inhibition rate of sterilized-AJ was only 27.10%, and the antifungal contribution rate of microorganisms was 46.48% (Figure 2C).

### Dynamic Changes in Pathogen, Antagonistic Microorganisms, Organic Acids and pH-Value During the Agricultural Jiaosu Inhibition Process

**FIGURE 3:**
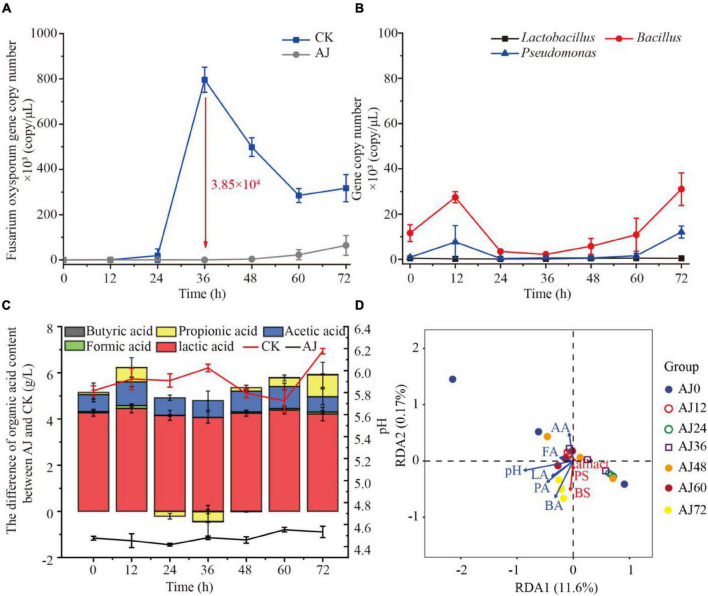
Dynamic changes observed in microorganisms and physicochemical factors during the inhibition process. **(A)** Dynamic changes in *Fusarium oxysporum* gene copy number; **(B)** dynamic changes in *Lactobacillus*, *Bacillus* and *Pseudomonas* gene copy number during AJ treatment; **(C)** dynamic changes of pH and differences in organic acids content between AJ and CK; **(D)** redundancy analysis of physicochemical factors and beneficial microorganism in AJ treatment during the inhibition process. LA, lactic acid; FA, formic acid; AA, acetic acid; PA, propionic acid; BA, butyric acid; BS, *Bacillus*; PS, *Pseudomonas*; LS, *Lactobacillus*; CK, without AJ treatment; AJ, with 10% AJ treatment. Data are mean ± standard deviation, *n* = 3.

To further reveal the antifungal mechanisms of AJ, three commonly antagonistic microbial genera (*Lactobacillus*, *Bacillus*, and *Pseudomonas*) were selected as representatives of AJ’s beneficial microorganisms. The qPCR results showed that AJ can effectively reduce the density of *F. oxysporum*, and the pathogen’s total abundance was approximately 21.14-fold higher in CK compared to the AJ treatment. The *F. oxysporum* gene copy number in CK rapidly increased from 4.93 × 10^1^ copy/μL at 0 h to 7.96 × 10^5^ copy/μL at 36 h (3.85 × 10^5^ copy/μL more than the level observed in the AJ treatment) (Figure 3A). Then, the dynamic changes in the antagonistic microorganisms in the AJ treatment were quantified. After 36 h, with the increase of *F. oxysporum*, the gene copy number of *Bacillus* and *Pseudomonas* increased rapidly, surpassing *Lactobacillus* and occupying the niche in AJ treatment. Among these genera, the density and increasing range of *Bacillus* were the largest (Figure 3B). By comparing the differences in organic acids between the AJ and CK treatments and the dynamic changes of pH value, it was found that the addition of AJ decreased the pH and promoted the accumulation of lactic and acetic acids (Figure 3C). In the redundancy analysis, among all the physicochemical properties, the arrow of pH were the longest, indicating that pH is the key predictor of antagonistic microorganism changes. Furthermore, *Lactobacillus*, *Bacillus*, and *Pseudomonas* were positively correlated with each other; Lactic, formic and butyric acids also had a positively relationship with antagonistic microorganism changes (Figure 3D).

### Isolation and Identification of Antagonistic Microbes

**FIGURE 4:**
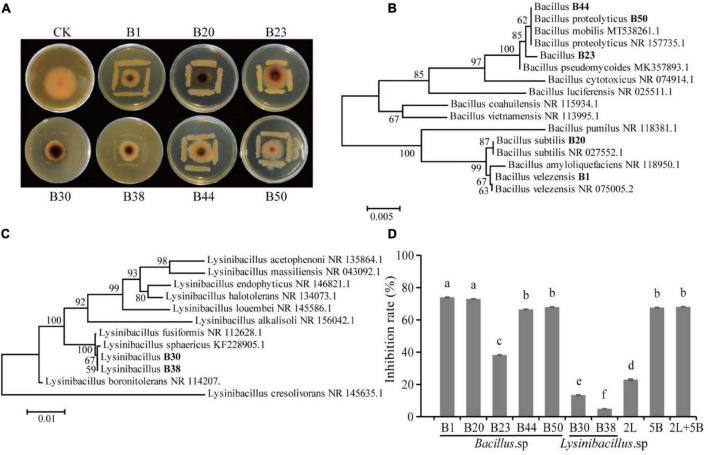
Isolation and identification of antagonistic microbes. **(A)** Antagonistic activity of isolates against *F. oxysporum*; **(B)** hierarchical clustering of *Bacillus* spp.; **(C)** hierarchical clustering of *Lysinibacillus* spp.; **(D)** antifungal effect of antagonistic strains alone or in combination against *F. oxysporum* (2L, equal proportion combination of two *Lysinibacillus* isolates; 5B, equal proportion combination of five *Bacillus* isolates; 2L + 5B, equal proportion combination of seven bacterial isolates). Values in the bar plot are expressed as the mean with standard deviation (*n* = 3). Different letters on the bars indicate statistically significant differences (*P* < 0.05) based on Duncan’s tests.

A total of 53 bacterial strains and 12 fungal strains were isolated from AJ. The inhibitory effect of all isolates was tested against *F. oxysporum* (values listed in Table 1), and a total of 47 isolates showed antagonistic activity against the *F. oxysporum*. Among them, the inhibition rates of isolates B1, B20, B23, B30, B38, B44, and B50 were above 20%, specifically, 43.16, 37.63, 24.21, 28.55, 28.95, 30.53, and 32.11%, respectively. Their antifungal activity is shown in Figure 4A, and the identified phylogenetic tree is shown in Figures 4B,C. The results indicate the presence of five *Bacillus* strains and two *Lysinibacillus* strains. Furthermore, the antifungal effect of antagonistic strains independently or in combination against *F. oxysporum* was determined. Figure 4D indicates that the antifungal effect of *Bacillus* spp. was significantly higher than *Lysinibacillus* spp. (*P* < 0.05), and B1 and B20 alone had the strongest antifungal effect (*P* < 0.05).

**TABLE 1:** Antifungal activity of 65 isolates against *Fusarium oxysporum.*

IsolatesID	Myceliumdiameter (mm)	Inhibition rate (%)	Isolates ID	Mycelium diameter (mm)	Inhibition rate (%)
B1	**24.00 ± 0.12^r^**	**43.16 ± 0.04^a^**	B34	36.75 ± 0.04^f–j^	2.89 ± 0.01^l^
B2	45.50 ± 0.09^de^	–	B35	36.25 ± 0.13^f–k^	4.47 ± 0.04^j–l^
B3	39.00 ± 0.87^d–f^	–	B36	38.00 ± 0.10^e–g^	–
B4	34.50 ± 0.09^g–m^	10.00 ± 0.03^e–l^	B37	33.00 ± 0.21^i–m^	14.74 ± 0.07^d–j^
B5	39.00 ± 0.17^d–f^	–	**B38**	**28.50 ± 0.15^n^** ^–^ **^q^**	**28.95 ± 0.05^bc^**
B6	34.50 ± 0.05^g–m^	10.00 ± 0.02^e–l^	B39	38.00 ± 0.21^e–g^	–
B7	32.67 ± 0.18^j–n^	15.79 ± 0.06^d–h^	B40	34.25 ± 0.29^g–m^	10.79 ± 0.09^e–l^
B8	46.50 ± 0.21^ab^	–	B41	32.00 ± 0.10^k–*o*^	17.89 ± 0.03^d–f^
B9	33.75 ± 0.13^g–m^	12.37 ± 0.04^e–l^	B42	45.00 ± 0.19^bc^	–
B10	37.25 ± 0.05^f–i^	1.32 ± 0.06^kl^	B43	35.50 ± 0.05^f–l^	6.80 ± 0.02^g–l^
B11	34.50 ± 0.05^g–m^	10.00 ± 0.02^e–l^	**B44**	**28.00 ± 0.10*^o^*** ^–^ **^q^**	**30.53 ± 0.03^bc^**
B12	31.50 ± 0.08^l–p^	19.47 ± 0.04^de^	B45	32.25 ± 0.15^k–n^	17.11 ± 0.05^d–f^
B13	32.00 ± 0.19^k–*o*^	17.89 ± 0.06^d–f^	B46	33.75 ± 0.38^g–m^	12.37 ± 0.12^e–l^
B14	33.75 ± 0.13^g–^**^m^**	12.37 ± 0.04^e–l^	B47	37.50 ± 0.25^e–h^	0.53 ± 0.08^kl^
B15	33.50 ± 0.15^h–m^	13.16 ± 0.05^e–k^	B48	32.50 ± 0.09^j–n^	16.32 ± 0.03^d–g^
B16	37.50 ± 0.21^e–h^	0.53 ± 0.07^kl^	B49	33.75 ± 0.11^g–m^	12.37 ± 0.03^e–l^
B17	32.75 ± 0.08^j–n^	15.53 ± 0.03^d–h^	**B50**	**27.50 ± 0.43^p^** ^–^ **^r^**	**32.11** ± 0.14^bc^
B18	42.75 ± 0.25^b–d^	–	B51	31.33 ± 0.05^l–p^	19.47 ± 0.02^de^
B19	46.50 ± 0.32^ab^	–	B52	33.00 ± 0.12^i–m^	14.74 ± 0.04^d–i^
B20	**25.75 ± 0.08^qr^**	**37.63 ± 0.03^ab^**	B53	32.75 ± 0.19^j–n^	15.53 ± 0.06^d–h^
B21	35.75 ± 0.13^f–l^	6.05 ± 0.04^h–l^	F1	31.50 ± 0.38**^d^**	13.27 ± 0.13^a^
B22	37.25 ± 0.28^f–i^	1.32 ± 0.09^i–l^	F2	31.00 ± 0.10**^d^**	14.97 ± 0.03^a^
B23	**30.00 ± 0.00^m^** ^–^ **^p^**	**24.21 ± 0.00^cd^**	F3	48.50 ± 0.15**^a^**	–
B24	42.50 ± 0.25^cd^	–	F4	33.00 ± 0.12**^cd^**	8.16 ± 0.04^a^
B25	34.50 ± 0.09^g–m^	10.00 ± 0.03^e–l^	F5	34.50 ± 0.50**^b^**^–^**^d^**	3.06 ± 0.17^a^
B26	35.50 ± 0.15^f–l^	6.84 ± 0.05^g–l^	F6	48.50 ± 0.35**^a^**	–
B27	49.25 ± 0.13^a^	–	F7	35.50 ± 0.36**^b^**^–^**^d^**	–
B28	34.50 ± 0.15^g–m^	10.00 ± 0.05^e–l^	F8	36.00 ± 0.10**^b^**	–
B29	34.00 ± 0.21^g–m^	11.58 ± 0.07^e–l^	F9	30.00 ± 0.00**^d^**	18.37 ± 0.00^a^
B30	**28.63 ± 0.20^n^** ^–^ **^q^**	28.55 ± 0.06^bc^	F10	32.00 ± 0.00**^d^**	11.56 ± 0.00^a^
B31	36.00 ± 0.10^f–k^	5.26 ± 0.03^i–l^	F11	37.00 ± 0.40**^b^**^–^**^d^**	–
B32	34.75 ± 0.04^f–l^	9.21 ± 0.01^f–l^	F12	39.50 ± 0.15**^bc^**	–
B33	37.75 ± 0.23^e–h^	–			

*Data are presented as means ± standard deviation. Different letters within each row indicate statistically significant differences (P < 0.05, n = 3) based on Duncan’s tests. The ANOVA of mycelium diameters of bacteria and fungi were analyzedseparately. “–” indicates no antifungal activity. Antifungal activity of the seven bacterial strains with the highest antagonistic ability are bolded.*

### Inhibition Effect of Agricultural Jiaosu and Isolated Strains in the Pot Experiment

**FIGURE 5:**
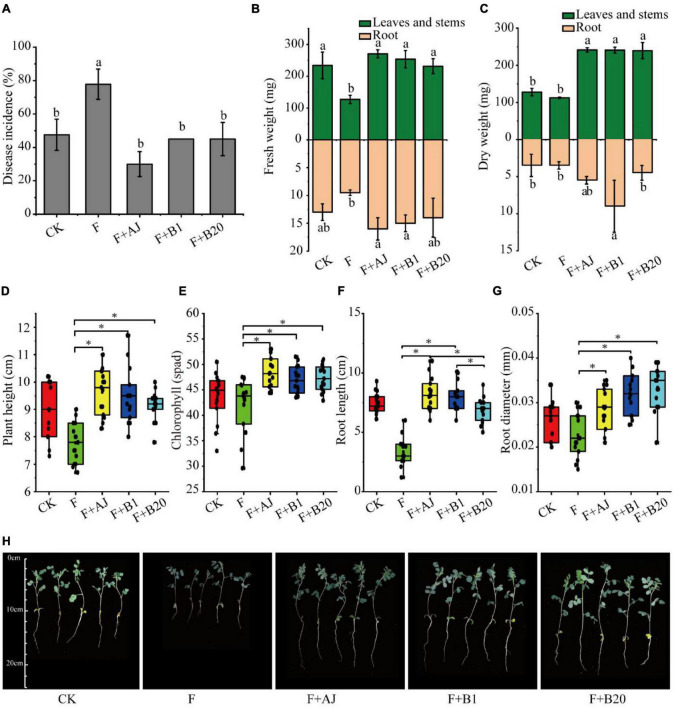
Disease incidence of root rot and growth indexes of *Astragalus*. **(A)** Disease incidence of root rot; **(B)** fresh weight; **(C)** dry weight; **(D)** plant height; **(E)** chlorophyll content; **(F)** root length; **(G)** root diameter; **(H)** growth states of *Astragalus* in different treatments. CK, no *F. oxysporum* and no biological control; F, *F. oxysporum* inoculation alone; F + AJ, *F. oxysporum* inoculation and 10% AJ control; F + B1, *F. oxysporum* inoculation and *B. velezensis* control; F + B20, *F. oxysporum* inoculation and *B. subtilis* control. Data are mean ± standard deviation, *n* = 15. Different letters and the symbol * on the bars indicate statistically significant (*P* < 0.05) differences based on Duncan’s tests.

In order to identify the effectiveness of AJ and antagonistic strains *in vivo*, the root rot disease incidence, plant height, chlorophyll content, root length, root diameter, fresh weight, and dry weight of *Astragalus* were further analyzed during the pot experiment. The experimental results showed that the application of AJ, or antagonistic strains B1 and B20 significantly reduced the disease incidence of root rot and promoted the growth of *Astragalus* (Figure 5). Compared with the F treatment, the disease incidence in F + AJ was reduced by 61.43% (*P* < 0.05), while the values for the F + AJ, F + B1, and F + B20 treatments were 30.00, 45.00, and 45.00%, respectively (Figure 5A). The F + AJ, F + B1, and F + B20 treatments determined a more significant increase in the fresh and dry weights of leaves and stems (Figures 5B,C), plant height (Figure 5D), chlorophyll content (Figure 5E) and root diameter (Figure 5F) of *Astragalus* compared to the F treatment (*P* < 0.05). Moreover, F + AJ and F + B1 treatments also produced a more significant increase in the fresh weight and length of roots compared the F treatment (Figures 5B,F) (*P* < 0.05).

## Discussion

*Fusarium* root rot is a critical factor restricting the sustainable production of medicinal plants. AJ as a promising biological agent gradually attracted attention. In the present study, it was found that the AJ fermented using medicinal plants above-ground was rich in beneficial microorganisms and metabolites such as organic acids, esters, alcohols, and that it can effectively inhibit the growth of *F. oxysporum*. In addition, beneficial microorganisms and organic acid metabolites may be the main driving factors of AJ’s antifungal action, and *B. subtilis* and *B. velezensis* were identified as the strongest antagonistic strains. Pot experiments confirmed that AJ and antagonistic strains can reduce the incidence of root rot and promote the growth of *Astragalus*.

### Agricultural Jiaosu’s Potential Capability to Suppress Soil-Borne Fungal Diseases

Biological control using beneficial microorganisms is a safe and effective method for suppressing soil-borne diseases (Mohamad et al., 2018). Previous studies have reported that AJ is a microbial ecosystem rich in beneficial microorganisms (such as *Lactobacillus* spp., *Pichia* spp., and others) and organic acids (such as lactic, acetic acids and others), and that it can effectively inhibit fungal pathogens (Rahman et al., 2020; Zhang et al., 2020). In this study, the waste leaves and stems of medicinal plants with anti-inflammatory effects were used as the fermentation material to enhance the antifungal activity of AJ. The MiSeq sequencing results showed that the core microbiome of AJ comprised Firmicutes, Proteobacteria, Bacteroidetes, and Actinobacteria. Among them, the relative abundance of Firmicutes and Proteobacteria was the highest (Figure 1A). These data are in line with previous reports, suggesting that Firmicutes and Proteobacteria dominate the entire bacterial community of AJ (Zhang et al., 2020). Previous studies have also shown that these two phyla were more abundant in healthy soils, they directly contributed to the pathogen inhibition (Huang et al., 2019; Wei et al., 2019), and AJ addition significantly increased the relative abundance of Firmicutes in organic fertilizers (Jiang et al., 2021). In regards to fungi, Ascomycota and Basidiomycota were the dominant phyla found in AJ (Figure 1B), and previous studies have also shown that these phyla have a higher relative abundance in soil after AJ irrigation (Wei et al., 2020). Moreover, in the present study, it was found AJ was rich in potential antagonistic microorganisms, such as *Lactobacillus*, *Pseudomonas*, *Burkholderia*, *Acinetobacter*, *Bacillus*, *Aspergillus*, *Pichia*, and *Penicillium* (Figures 1C,D), and numerous previous studies indicated that they can control soil-borne pathogens through competition, antibiosis, parasitism, and induced systemic resistance, which have been widely used to protect crops from disease (Van Wees et al., 2008; Usman et al., 2013; Gao et al., 2021).

Furthermore, microbial metabolites containing many antifungal compounds, also play an important role in biological control. These metabolites are easy to decompose and coexist in the environment, and they can also promote interactions and communication between organisms, improve plant growth, induce systemic resistance, and inhibit the growth of pathogenic fungi (Gao et al., 2017). The fermentation of AJ is a process in which microorganisms decompose sugar and organic materials, and metabolites gradually accumulate, especially organic acids. The results of this study showed that AJ had a low pH value, and was rich in organic acids (such as acetic, formic and butyric acids). It has been proved that organic acids can inhibit soil-borne fungi, such as *F. oxysporum*, by acidifying the environment and changing the cell membrane permeability of pathogen (Momma et al., 2006). Accumulating evidence suggests that VOCs can act as antimicrobial agents, and are important for the regulation of the interactions between pathogens and beneficial microorganisms (Raza et al., 2021). GCMS analysis identified 29 unique VOCs in the AJ, consisting mainly of esters (11) and alcohols (8) (Supplementary Table 5). In this study, the inhibitory effect of AJ on *F. oxysporum* was studied for the first time, and it was confirmed that, the higher the dose of AJ is, the stronger the inhibition of *F. oxysporum* (Figure 2B). This is consistent with the results we previously reported (Zhang et al., 2020), which indicated that AJ can effectively inhibit plant pathogenic fungi, and that the antifungal activity is positively correlated with dosage. All these results suggest that AJ has a great potential capability to suppress soil-borne fungal diseases, and its application will be a promising strategy for disease control.

### Main Driving Factors of Agricultural Jiaosu Controlling *Fusarium* Root Rot

The AJ is a complex fermentation solution (Arun and Sivashanmugam, 2015, 2017), and its antifungal activity is caused by many factors. However, the main antifungal factor of AJ has not been reported in the literature. Our results indicate that AJ’s microbial components were critical for the effective suppression of *Fusarium* root rot, and its antifungal contribution rate was 46.48% (Figure 2C), indicating that these microbial components may be the main driving factor of AJ’s antifungal activity. Interestingly, it was found that the population densities of *Bacillus* and *Pseudomonas* genus increased rapidly and occupied a niche in the dynamic inhibition process induced by the AJ treatment, and the density and increasing range of *Bacillus* were the largest (Figure 3B). At the same time, the gradual accumulation of organic acid metabolites—especially lactic acid and acetic acid—lead to a decreased in the pH value (Figure 3C). Previous studies have reported that the beneficial microbes suppress fungal diseases by forming organic acids, which in turn lower the environmental pH (Yu et al., 2019). Redundancy analysis revealed that such microorganisms are positively correlated with lactic, formic, butyric acids, and pH value, and that the population densities of *Bacillus*, *Pseudomonas* and *lactobacillus* are correlated with each other (Figure 3D). These data imply that the combined effects of beneficial microbes (such as *Bacillus*, *Pseudomonas* and *Lactobacillus*) and organic acid metabolites (such as lactic, acetic, formic, and butyric acids) may represent the main driving factors and biocontrol mechanism through which AJ effectively suppresses *Fusarium* root rot.

Further isolation and identification of antagonistic strains revealed 47 AJ’s strains with antagonistic activity against *F. oxysporum*. Among seven isolates with the highest antifungal activity, five isolates belonged to the *Bacillus* genus. In particular, *B. subtilis* and *B. velezensis* were identified as the most effective antagonistic strains (Figure 3 and Table 1). A number of well-known fungal antagonists belong to this genus, which has also been identified as the dominant genus in organic fertilizers treated by AJ treatment (Jiang et al., 2021). Among the *Bacillus* species, the above-mentioned *B. subtilis* and *B. velezensis* are ideal for the formulation of biocontrol agents, and can provide resistance against many pathogens by producing spores, biofilms and antibiotic substances (Cho et al., 2018; Charron-Lamoureux et al., 2020). In this study, the pot experiment confirmed that AJ treatments can significantly suppress *Fusarium* root rot; in fact, the disease incidence was 61.43% lower than that observed in the F treatment. Moreover, AJ treatments significantly increased the plant height, root length, root diameter, chlorophyll content, and the fresh and dry weights of *Astragalus* (Figure 5). Although the *B. subtilis* and *B. velezensis* isolates also significantly reduced the incidence of root rot, their control efficacy was slightly lower than that obtained in the AJ treatment (Figure 5A). It is well known that the performance of an individual antagonist may be unstable under different environmental conditions, and this applies also to *B. subtilis* and *B. velezensis* (Kalantari et al., 2018).

### Agricultural Jiaosu Can Solve the Bottleneck of the High-Cost of Biological Agents Through Resource Recycling Patterns

**FIGURE 6:**
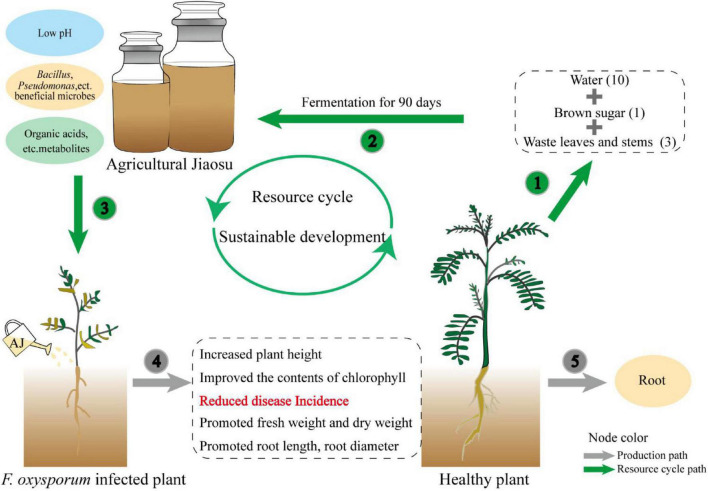
Conceptual model illustrating how the application of AJ can establish a whole plant recycling pattern to promote the sustainable development of medicinal plants.

The effectiveness of antagonists is generally accepted, however, their wide application is limited by their high cost and instability. The preparation and storage of antagonistic microorganisms require strict aseptic conditions and high requirements in terms of technology and equipment, which undoubtedly represent a huge economic investment. In contrast, AJ is a low-cost and easy-to-operate mixed microbial fermentation technique. It is easily prepared by the fermenting organic waste, sugar and water (Rani et al., 2020), and it does not require expensive equipment. Its lower pH reduces the risk of contamination during the fermentation process (Bartkiene et al., 2020), and our research has proved that it has a strong disease suppression capability. Therefore, this novel biotechnology can solve the bottleneck of the high cost of biological agents (Zhang et al., 2020). As shown in the conceptual model (Figure 6), the waste stems and leaves of medicinal plants can be used to prepare an AJ with a low pH value, beneficial microorganisms, and metabolites, which then can function as a biocontrol agent to suppress *Fusarium* root rot, and promote the growth of medicinal plants. The whole plant recycling pattern can convert agricultural waste into biological agents, promote the recycling of waste resources back into the soil, and conducive to the sustainable development of agriculture (Arun and Sivashanmugam, 2015). Thus, AJ can be used as a low-cost, stable, and practical biological agent to replace fungicides and expensive antagonists.

## Conclusion

The results of this study showed that the AJ fermented using medicinal plants above-ground had a low pH value, and was rich in beneficial microorganisms, and metabolites, such as organic acid, esters and alcohols, etc. AJ can effectively inhibit *F. oxysporum* by the combined effects of beneficial microbes (such as *Bacillus*, *Pseudomonas*, and *Lactobacillus*) and organic acid metabolites (such as lactic acid, formic acid, and butyric acid). The application of AJ and *Bacillus* spp., isolated from AJ, can significantly suppress *Fusarium* root rot, and promote the growth of *Astragalus membranaceus*. These results suggest that AJ should be considered as a cost-effective way to manage soil-borne diseases and promote agricultural sustainable development.

## Data Availability Statement

The original contributions presented in the study are included in the article/Supplementary Material, further inquiries can be directed to the corresponding author.

## Author Contributions

YG, YZ, and XFW conceived and designed the experiments. YG and XC performed the experiments. YZ and XC contributed reagents, materials, and analysis tools. YG, ZZ, and YZ analyzed the data. YG wrote the manuscript. XFW, XHW, XD, ZZ, and YH revised the manuscript. All authors approved the final version of the article for publication.

## Conflict of Interest

The authors declare that the research was conducted in the absence of any commercial or financial relationships that could be construed as a potential conflict of interest.

## Publisher’s Note

All claims expressed in this article are solely those of the authors and do not necessarily represent those of their affiliated organizations, or those of the publisher, the editors and the reviewers. Any product that may be evaluated in this article, or claim that may be made by its manufacturer, is not guaranteed or endorsed by the publisher.

## Footnotes

1
http://www.arb-silva.de

2
http://unite.ut.ee/index.php

## Supplementary Material

Supplementary Figure 1 Research roadmap.

Supplementary Figure 2 The half-maximal inhibitory concentration (IC50) of AJ.

Supplementary Table 1 Amplification primers used in this study.

Supplementary Table 2 Processed sample data information to analyze bacterial and fungal community of AJ.

Supplementary Table 3 Alpha diversity of bacterial and fungal community of AJ.

Supplementary Table 4 The relative abundance of top 10 bacterial and fungal genus.

Supplementary Table 5 Volatile organic compounds (VOCs) of AJ.
