# Supplementary figures and images for: Agricultural Jiaosu: An Eco-Friendly and Cost-Effective Control Strategy for Suppressing Fusarium Root Rot Disease in Astragalus membranaceus
Source: Front Microbiol. 2022 Mar 31;13:823704. doi: 10.3389/fmicb.2022.823704 (PMC9008360; doi:10.3389/fmicb.2022.823704)

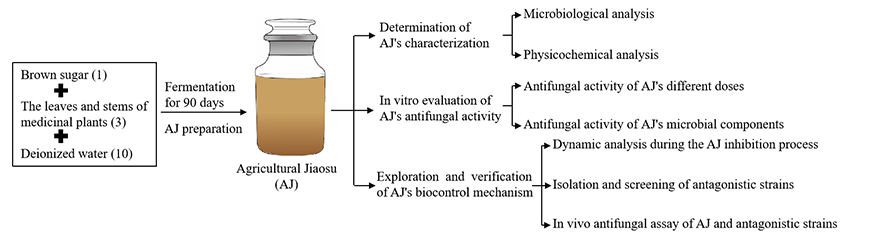

Supplement: Supplementary Figure 1 — Research roadmap. [file Image_1.TIF]

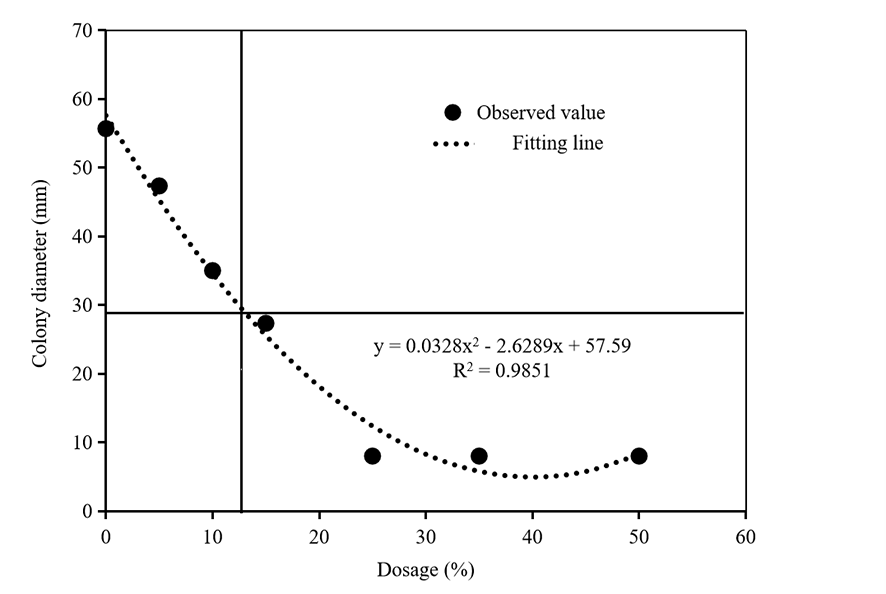

Supplement: Supplementary Figure 2 — The half-maximal inhibitory concentration (IC50) of AJ. [file Image_2.TIF]
