# Supplementary material for: Agricultural Jiaosu: An Eco-Friendly and Cost-Effective Control Strategy for Suppressing Fusarium Root Rot Disease in Astragalus membranaceus
Source: Front Microbiol. 2022 Mar 31;13:823704. doi: 10.3389/fmicb.2022.823704 (PMC9008360; doi:10.3389/fmicb.2022.823704)
Supplement: Supplementary Table 1 — Amplification primers used in this study. [file Table_1.DOC]

**Supplementary Table 1.** Amplification primers used in this study

| **Target genes** | **Primers** | **Sequence (5′-3′)** | **Reference** |
| --- | --- | --- | --- |
| *Fusarium oxysporum* | EF1H | ATGGGTAAGGAAGACAAGAC | Fen Gao et al. (2018) |
|  | EF2T | AAGTACCAGTGATCATGTT |  |
| *Bacillus* | BacF | GGGAAACCGGGGCTAATACCGGAT | Koji Mori et al. (2004) |
|  | BacR | GTCACCTTAGAGTGCCC |  |
| *Lactobacillus* | F_alllact_IS | TGGATGCCTTGGCACTAGGA | Monique Haarman  et al. (2006) |
|  | R_alllact_IS | AAATCTCCGGATCAAAGCTTACTTAT |  |
| *Pseudomonas* | Ps-for | GAGTTTGATCCTGGCTCAG | Paolina Garbeva  et al. (2004) |
|  | Ps-rev | CCTTCCTCCCAACTT |  |
