# Supplementary material for: Agricultural Jiaosu: An Eco-Friendly and Cost-Effective Control Strategy for Suppressing Fusarium Root Rot Disease in Astragalus membranaceus
Source: Front Microbiol. 2022 Mar 31;13:823704. doi: 10.3389/fmicb.2022.823704 (PMC9008360; doi:10.3389/fmicb.2022.823704)
Supplement: Supplementary Table 2 — Processed sample data information to analyze bacterial and fungal community of AJ. [file Table_2.DOC]

**Supplementary Table 2.** Processed sample data information to analyze Bacterial and fungal community of AJ

|  | **Bacterial** | **Fungal** |
| --- | --- | --- |
| Raw Reads | 280874 | 347512 |
| Clean Reads | 277892 | 345815 |
| AvgLen | 424.89 | 235.50 |
| Number of sequences | 232501 | 331457 |
| Number of ASVs | 4262 | 587 |
