# Supplementary material for: Agricultural Jiaosu: An Eco-Friendly and Cost-Effective Control Strategy for Suppressing Fusarium Root Rot Disease in Astragalus membranaceus
Source: Front Microbiol. 2022 Mar 31;13:823704. doi: 10.3389/fmicb.2022.823704 (PMC9008360; doi:10.3389/fmicb.2022.823704)
Supplement: Supplementary Table 3 — Alpha diversity of bacterial and fungal community of AJ. [file Table_3.DOC]

**Supplementary Table 3.** Alpha diversity of Bacterial and fungal community of AJ

|  | **Bacterial** | **Fungal** |
| --- | --- | --- |
| Chao1 | 1463.1677 | 199.6624 |
| Shannon | 5.9194 | 3.4025 |
| Simpson | 0.9331 | 0.6110 |
| Pielou’s evenness | 0.6086 | 0.4394 |
| Good’s coverage | 0.9975 | 0.9998 |
