# Supplementary material for: Agricultural Jiaosu: An Eco-Friendly and Cost-Effective Control Strategy for Suppressing Fusarium Root Rot Disease in Astragalus membranaceus
Source: Front Microbiol. 2022 Mar 31;13:823704. doi: 10.3389/fmicb.2022.823704 (PMC9008360; doi:10.3389/fmicb.2022.823704)
Supplement: Supplementary Table 4 — The relative abundance of top 10 bacterial and fungal genus. [file Table_4.DOC]

**Supplementary Table 4.** The relative abundance of top 10 Bacterial and fungal genus

| **Bacterial** | |  | **Fungal** | |
| --- | --- | --- | --- | --- |
| **Name** | **Relative abundance**  **(%)** |  | **Name** | **Relative abundance**  **(%)** |
| *Lactobacillus* | 52.35 |  | *Aspergillus* | 32.28 |
| *Halomonas* | 7.50 |  | *Tausonia* | 25.88 |
| *Pelagibacterium* | 4.06 |  | *Phaeoisaria* | 6.01 |
| *Hydrogenibacillus* | 3.90 |  | *Lecanicillium* | 3.28 |
| *Prevotella*_1 | 3.57 |  | *Verticillium* | 2.68 |
| *Muribaculaceae* | 3.18 |  | *Cutaneotrichosporon* | 1.57 |
| *Ralstonia* | 1.04 |  | *Pichia* | 0.83 |
| *Ruminococcaceae*_NK4A214_group | 1.01 |  | *Candida* | 0.82 |
| *Alistipes* | 0.78 |  | *Malassezia* | 0.70 |
| *Succiniclasticum* | 0.75 |  | *Fusarium* | 0.52 |
| Others | 21.87 |  | Others | 25.43 |
