# Supplementary material for: Agricultural Jiaosu: An Eco-Friendly and Cost-Effective Control Strategy for Suppressing Fusarium Root Rot Disease in Astragalus membranaceus
Source: Front Microbiol. 2022 Mar 31;13:823704. doi: 10.3389/fmicb.2022.823704 (PMC9008360; doi:10.3389/fmicb.2022.823704)
Supplement: Supplementary Table 5 — Volatile organic compounds (VOCs) of AJ. [file Table_5.DOC]

**Supplementary Table 5.** Volatile organic compounds (VOCs) of AJ

| **S no.** | **Retention time (min)** | **Compound** | **Matching degree** | **Area**  **(%)** |
| --- | --- | --- | --- | --- |
| 1 | 6.27 | 3-Heptanol, 6-methyl- | 72 | 14.00 |
| 2 | 12.01 | Phenol, 4-ethyl-2-methoxy- | 91 | 9.07 |
| 3 | 10.56 | L-.alpha.-Terpineol | 50 | 7.85 |
| 4 | 5.78 | 1-Octen-3-ol | 78 | 7.61 |
| 5 | 10.62 | Methyl salicylate | 51 | 6.84 |
| 6 | 13.18 | Phenol, 2-methoxy-6-(2-propenyl)- | 83 | 4.80 |
| 7 | 1.01 | 1-Butanol, 3-methyl-, acetate | 78 | 4.79 |
| 8 | 14.4 | Ethanone, 1-(2-hydroxy-4-methoxyphenyl)- | 90 | 4.31 |
| 9 | 11.63 | Acetic acid, 2-phenylethyl ester | 90 | 3.66 |
| 10 | 9.21 | Phenylethyl Alcohol | 91 | 3.31 |
| 11 | 9.29 | Phenylethyl Alcohol | 83 | 3.11 |
| 12 | 10.2 | Benzoic acid, ethyl ester | 91 | 2.80 |
| 13 | 8.83 | 1,6-Octadien-3-ol, 3,7-dimethyl- | 78 | 2.69 |
| 14 | 11 | Bicyclo[4.1.0]hept-2-ene | 53 | 2.47 |
| 15 | 6.36 | Hexanoic acid, ethyl ester | 47 | 2.33 |
| 16 | 11.44 | Benzeneacetic acid, ethyl ester | 91 | 2.01 |
| 17 | 10.97 | Tricyclo[3.3.0.0(2,8)]octan-3-one, 8-methyl- | 59 | 1.18 |
| 18 | 7.59 | Benzyl alcohol | 62 | 1.15 |
| 19 | 10.38 | Butanedioic acid, diethyl ester | 83 | 1.04 |
| 20 | 10.89 | Phenol, 3-ethyl- | 49 | 0.97 |
| 21 | 1.12 | Propanoic acid, 2-methyl-, 2-methylbutyl ester | 64 | 0.90 |
| 22 | 11.87 | Benzoic acid, 2-hydroxy-, ethyl ester | 80 | 0.90 |
| 23 | 8.69 | Mequinol | 58 | 0.87 |
| 24 | 7.13 | Phenol | 64 | 0.79 |
| 25 | 13.02 | Benzenepropanoic acid, ethyl ester | 74 | 0.72 |
| 26 | 11.19 | Octa-2,4,6-triene | 49 | 0.66 |
| 27 | 13.73 | 2,6-Dimethoxybenzonitrile | 59 | 0.61 |
| 28 | 1.45 | Ethanol | 58 | 0.46 |
| 29 | 10.09 | Acetic acid, phenylmethyl ester | 80 | 0.40 |
